# *In vivo* characterisation of the *Vibrio vulnificus* stressosome: A complex involved in reshaping glucose metabolism and motility regulation, in nutrient- and iron-limited growth conditions.

**DOI:** 10.1016/j.crmicr.2023.100186

**Published:** 2023-03-02

**Authors:** Laura Cutugno, Borja Khatabi Soliman Tamayo, Piet N.L. Lens, Conor O'Byrne, Jan Pané-Farré, Aoife Boyd

**Affiliations:** a School of Natural Sciences, University of Galway, Ireland; b School of Biological and Chemical Sciences, University of Galway, Ireland; c Centre for synthetic Microbiology (SYNMIKRO) & Department of Chemistry, Philipps-University Marburg, Germany

**Keywords:** Vibrio vulnificus, Stress response, Stressosome, Iron metabolism, Virulence, Motility

## Abstract

Stressosomes are signal-sensing and integration hubs identified in many bacteria. At present, the role of the stressosome has only been investigated in Gram-positive bacteria. This work represents the first *in vivo* characterisation of the stressosome in a Gram-negative bacterium, *Vibrio vulnificus*. Previous *in vitro* characterisation of the complex has led to the hypothesis of a complex involved in iron metabolism and control of c-di-GMP levels. We demonstrate that the stressosome is probably involved in reshaping the glucose metabolism in Fe- and nutrient-limited conditions and mutations of the locus affect the activation of the glyoxylate shunt. Moreover, we show that the stressosome is needed for the transcription of *fleQ* and to promote motility, consistent with the hypothesis that the stressosome is involved in regulating c-di-GMP. This report highlights the potential role of the stressosome in a Gram-negative bacterium, with implications for the metabolism and motility of this pathogen.

## Highlights

• Bacterial stressosome controls glucose metabolic pathways.
• Stressosome regulates motility of pathogenic *vibrio vulnificus*.
• Association of vibrio stressosome with 2-component regulatory system.

## Graphical abstract

**Figure:**
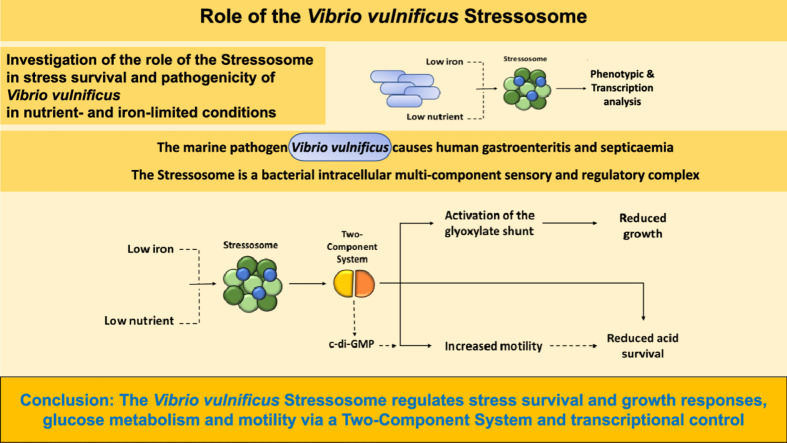

## 1 Introduction

In order to survive and thrive, bacteria need to monitor their environment continuously and respond efficiently when changes are encountered. In the case of pathogenic bacteria, such changes occur during infection and require an appropriate response to guarantee successful colonisation of the host (Fang et al., 2016). One critical step of the stress response in bacteria is stress-sensing and signal integration and one widely conserved signalling complex in bacteria that achieves this is the high-molecular weight complex known as the stressosome. The stressosome is found in *Bacillus subtilis* (Delumeau et al., 2006; Chen et al., 2003; Kim et al., 2004) and other Gram-positive bacteria, including the food-borne pathogen L. *monocytogenes* (Williams et al., 2019; Dessaux et al., 2020; Dessaux et al., 2021), as well as some archaeal species (Pané-Farré et al., 2017).

The stressosome in *B. subtilis* consists of a core containing several copies of the RsbR and RsbS proteins and a mobile protein with kinase function, RsbT, that binds to the stressosome core in the absence of stimuli and is released, following phosphorylation of the core proteins, when stress is sensed (Marles-Wright et al., 2008; Kwon et al., 2019). RsbR is the protein that senses the stress signal, through its N-terminal sensory domain (Murray et al., 2005). In both *B. subtilis* and L. *monocytogenes,* several RsbR paralogues are present but the activation signal of only YtvA and its orthologs has been identified (Gaidenko et al., 2006; Avila-Pérez et al., 2006). In these organisms, the release of RsbT from the core stressosome leads, through a phosphorylation cascade, to the release of the alternative sigma factor B (SigB or σ^B^) from its anti-sigma factor RsbW (Yang et al., 1996; Gaidenko et al., 1999; Eymann et al., 2011). The binding of SigB to the core RNA-polymerase promotes the transcription of a large set of genes involved in stress response (Petersohn et al., 1999; Guerreiro et al., 2020). The stressosome genetic locus (*rsbR-rsbS-rsbT*) is widely distributed amongst bacteria (Pané-Farré et al., 2017; Pané-Farré et al., 2005) and it was previously identified in a large number of Gram-negative species. This locus has been found in bacteria belonging to the phyla Cyanobacteria, Bacteroides and Proteobacteria (α, β, γ) (Pané-Farré et al., 2005), thus showing a significant prevalence amongst Gram-negative bacteria. Interestingly many of these species do not possess SigB. Instead, a large variety of downstream putative effectors, chromosomally associate with the *rsbR-rsbS-rsbT* locus has been identified. These include alternative sigma factors, secondary messengers and two-component signalling (Pané-Farré et al., 2017; Pané-Farré et al., 2005). Despite this high variability and the abundance of Gram-negative strains carrying the stressosome locus, the *in vivo* role of the complex in a Gram-negative bacterium with a different downstream module has never been investigated.

An example of such a complex stressosome locus is present in the Gram-negative human pathogen *Vibrio vulnificus* (Oliver, 2006). *V. vulnificus* can infect the host through consumption of raw or undercooked seafood or following exposure of wounds to contaminated seawater (Baker-Austin and Oliver, 2018; Heng et al., 2017; Jones and Oliver, 2009; Linkous and Oliver, 1999; Oliver, 2015; Oliver, 2005). *V. vulnificus* can cause severe infection, often with fatal outcomes, especially in patients with underlying health conditions that cause a higher blood level of iron (Wright et al., 1981; Bullen et al., 1991). Our analysis showed that the stressosome locus is present in 44% of sequenced *V. vulnificus* strains. Of the 720 *V. vulnificus* complete and draft genome sequences in the NCBI database at the time of analysis 317 possess the stressosome locus with most of these strains belonging to Lineages I and 3. It consists of an upstream module containing the stressosome genes *VvrsbR, VvrsbS, VvrsbT* and *VvrsbX* (homologous of the *rsbR, S, T* and *X* in *B. subtilis*) and a downstream module containing two genes (*VvD1* and *VvD2*) encoding a two-component regulatory system (TCS), probably involved in regulating the intracellular level of cyclic-di-GMP (c-di-GMP) (Pané-Farré et al., 2017; Pané-Farré et al., 2005). Transcriptional studies in *V. vulnificus* have previously reported an upregulation of these stressosome genes in conditions mimicking the bacterial natural environment, in the absence of oxygen, and in Artificial Sea Water (ASW) (Williams et al., 2014; Morrison et al., 2012; Phippen and Oliver, 2017). More recently, *in vitro* characterisations of the stressosome in *V. brasiliensis* and *V. vulnificus* have led to the hypothesis that this is an oxygen- and iron-dependant signalling hub, and provided important insights into the structure and geometry of the stressosome in Gram-negative bacteria (Jia et al., 2016; Heinz et al., 2022). Despite these results, *in vivo* characterisation of the complex in *V. vulnificus* has been limited to date, with the exception of proteomic analysis of a stressosome upstream mutant, *V. vulnificus* ΔRSTX, that led to the hypothesis that the stressosome is involved in iron sensing (Heinz et al., 2022).

Here, we describe the physiological and transcriptional effects of the deletion of both the upstream and downstream modules of the stressosome operon in *V. vulnificus*. The two mutants produce similar phenotypes indicating that there is a functional link between the two modules. We demonstrate that the stressosome is expressed and is active in a nutrient-limited environment. We also show that the stressosome is most likely involved in remodelling metabolism in *V. vulnificus* in this environment, through activation of the glyoxylate shunt, an alternative to the TCA cycle that allows acclimation to low iron/nutrient conditions in several microorganisms (Koedooder et al., 2018; Kornberg, 1966). We also find that the stressosome is involved in the motility of *V. vulnificus,* probably through a c-di-GMP-mediated regulation of the motility regulator *fleQ* (Jyot et al., 2002; Matsuyama et al., 2016)*.* Surprisingly no positive effects of the stressosome on stress response were detected and instead the stressosome mutants were found to be more resistant to acid stress. This work shows that the stressosome in Gram-negative bacteria is functionally different from stressosomes present in Gram-positive bacteria and highlights its role in remodelling metabolism to cope with nutritional and iron limitation.

## 2 Materials and methods

### 2.1 Strains, plasmids and growth conditions

The clinical isolate *Vibrio vulnificus* CMCP6 (Kim et al., 2003) was used as the parental strain for the construction of the two stressosome mutants in this work (see below). The auxotrophic *E. coli* β2163 was used to perform bacterial conjugation (Demarre et al., 2005). *Vibrio vulnificus* was cultured in Luria Bertani (LB) medium with additional 0.4 M NaCl (LBN) or Chemically Defined Medium (CDM) (9.94 mM Na_2_HPO_4_, 10.03 mM KH_2_PO_4_, 0.81 mM MgSO_4_∙7H_2_O, 9.35 mM NH_4_Cl, 856 mM NaCl, 7.5 mM α-D(+)-Glucose, 0.75 μM FeCl_3_) at 37 °C. The FeCl_3_ stock was prepared in 1 M HCl. *E. coli* β2163 was grown in LB medium supplemented with 0.3 mM 2,6-Diaminopimelic acid (DAP) at 37 °C. Overnight broth cultures were grown in 2 ml or 20 mL medium, in 15 mL bacterial culture tubes and 250 mL flasks, respectively, at 37 °C with agitation at 150 rpm. Specific growth conditions, different from the one indicated above, are described in the appropriate sections. All chemicals and reagents were supplied by Sigma-Aldrich, unless indicated otherwise.

### 2.2 Construction of the *V. vulnificus* stressosome knock-out mutants

Two knock-out mutants were used in this work, one lacking the upstream module of the stressosome locus consisting of the *VvrsbR, VvrsbS, VvrsbT* and *VvrsbX* genes (*V. vulnificus* ΔRSTX) and one lacking the first gene of the downstream module *VvD1* (*V. vulnificus* ΔD1), encoding for the sensor kinase of a two-component regulatory system, predicted to be involved in cyclic-di-GMP regulation. Construction of the upstream ΔRSTX mutant has been previously described (Cutugno et al., 2022). Construction of the downstream ΔD1 mutant was performed using the same protocol. Briefly, the downstream knockout cassette (Eurofins) contained a deletion from nucleotide 12 to nucleotide 3185 of *VvD1*. It was digested with *Sbf*I and *Sph*I and cloned into the pDS132 suicide vector (Philippe et al., 2004), between the *sacB* promoter and the *lac* operator, resulting in the knockout plasmid pDS_ΔD1. This was transformed by electroporation into *E. coli* β2163 and transformants were selected on LB + 25 μg/mL Chloramphenicol + 0.3 mM DAP agar plates. The recombinant *E. coli* strain was used as the donor in bi-parental conjugation to transfer the plasmid into the recipient *V. vulnificus* CMCP6. The auxotrophic *E. coli* β2163 (Demarre et al., 2005) allows counter-selection of first recombinants after conjugation and avoids previously reported pleiotropic effects associated with *V. vulnificus* rifampicin-resistant strains, which had been typically used for this purpose during genetic manipulation of this species (Cutugno et al., 2020). *V. vulnificus* CMCP6 and *E. coli* β2163(pDS_ΔD1) were grown overnight at 37 °C in LBN and LB + 25 μg/mL Chloramphenicol + 0.3 mM DAP, respectively. Cultures were washed by centrifugation in LB broth, the two strains were mixed at a 1:1 ratio (v/v), spotted onto LB + 0.3 mM DAP agar plates and incubated for 7 h at 37 °C. *V. vulnificus* transconjugants carrying the knock-out plasmid integrated into the chromosome by homologous recombination were selected on LBN + 5 μg/mL Chloramphenicol. In order to promote the excision of the plasmid from the chromosome of *V. vulnificus,* the second event of homologous recombination was favoured by culturing the first recombinant cells in LBN broth without selective antibiotic. Second recombinant cells were selected on LBN + 5% sucrose (w/v) because the *sacB* gene present in the suicide vector pDS132 does not allow growth in the presence of sucrose and growth on this media is a sign of a successful excision of the plasmid from the bacterial genome. This event can equally generate wild-type cells or deletion mutants. For this reason, mutants were selected by PCR screening of colonies grown on LBN + 5% sucrose using Taq polymerase (Bioline) and the primers D1_For (5′-GATGGGGCTGTCATTCACGA-3′) and D1_Rev (5′-TCATGCAAAGGGGCAGACTT-3′). PCR products of different sizes for the mutant and the wild-type were visualised through agarose gel electrophoresis. One mutant was selected and Whole Genome Sequencing (WGS) analysis was performed. Genomic DNA was extracted using the Wizard Genomic DNA Purification Kit (Promega), according to the manufacturer's protocol. Genomic DNA was sequenced by MicrobesNG (Birmingham, UK) using Illumina technology. The average read length was 190 nucleotides for each sample and average fold coverage was 100. BreSeq version 0.32.0 was used to call base substitution mutations Read Alignment evidence using Consensus mode, with a mutation E-value cut-off of 10 and frequency cut-off of 0.8 (80%).

### 2.3 Growth characterisation

Growth was assessed for all the strains at 37 °C in CDM and LBN. The strains were grown overnight in 20 mL LBN at 37 °C with at least three biological replicates. Bacterial cultures were then washed and diluted in 30 mL of appropriate media to a final OD_600_ of 0.05 in 250 mL flasks. The flasks were incubated at 37⁰C with 150 rpm agitation. OD_600_ was measured every 60 min for the first 12 h and at different time points (as indicated in Figures) between 24 and 72 h.

### 2.4 High-performance liquid chromatography (HPLC) analysis

In order to study the glucose depletion and organic acid accumulation in the media during growth of *V. vulnificus* WT and stressosome mutants in CDM at 37 °C, 1 mL culture was taken from the culture flask at different time points and filtered with a 0.22 µm filter for downstream HPLC analysis. Samples were stored in a tightly closed tube at −20 °C. The HPLC analysis was performed in a liquid chromatograph (LC) (1260 Infinity II, Agilent, USA) with a refractive index detector (RID) and a Hi-Plex H column (300 × 7.7 mm) at 60 °C. 5 mM H_2_SO_4_ was used as mobile phase and the flow rate was set at 0.4 mL/min. For each time point three replicates were analysed.

### 2.5 RNA extraction and qRT-PCR gene transcription analysis

For RNA extraction, cell culture aliquots were taken from wild-type, ΔRSTX and ΔD1 overnight cultures in LBN and after 6, 12 and 24 h growth in CDM. One volume of bacterial culture, containing approximately 0.5 OD_600_ of cells, was mixed with 2 vol of RNAprotect (Qiagen) and processed according to the manufacturer's instructions. Treated pellets were stored for a maximum of 24 h at −20 °C. Before RNA extraction, each pellet was resuspended in 200 µL TE buffer (30 mM Tris–HCl, 1 mM EDTA, pH 8.0) + 15 mg/mL lysozyme + 10 µL ready-to-use QIAGEN Proteinase K (20 mg/mL) and incubated 10 min at room temperature to lyse the bacterial cells. Lysates were used for RNA extraction with RNeasy Mini Kit (Qiagen) following the manufacturer's protocol. RNA was eluted in 50 µL RNase-free water and a rigorous DNase treatment was performed using the TURBO DNA-free kit (Life Technologies), according to the manufacturers’ instruction, to remove traces of DNA. RNA concentration and quality were assessed with a NanoDrop spectrophotometer and only samples with a 260/280 ratio ≥ 1.7 were used for the qRT-PCR protocol, and RNA integrity was verified on a 1.5% (w/v) agarose gel. cDNA was prepared from 1 µg total RNA using the Transcriptor First Strand cDNA Synthesis Kit (Roche) and Random Hexamer Primer contained in the kit. The RT reaction was carried out using a 10 min incubation at 25 °C followed by 60 min at 50 °C. The primers for the qPCR reactions (Table 1) were designed using Primer-BLAST (Ye et al., 2012) and primer specificity and efficiency calculated. 2 µL of a 1:10 dilution of the cDNA were used in a 10 µL reaction to study transcription of the selected gene targets through qPCR, using the SYBR Green I Master (Roche) and the LightCycler 480 (Roche). Each reaction was tested in two technical replicates. The transcription of each gene was normalised by using the *tuf* gene (elongation factor Tu) as endogenous control, similarly to previously-published transcriptional studies in *V. vulnificus* (Nowakowska and Oliver, 2013), and fold changes in transcription levels were calculated using the Pfaffl method (Pfaffl, 2001).

**Table 1:** Primers for qRT-PCR on stressosome, metabolism, stress response and motility target genes in V. vulnificus CMCP6 wild-type and stressosome mutants.

Gene	Sequence (5′->3′)
*tuf* (VV1_1203)	Forward: AAGTTTACGGCGGTGCTGCT Reverse: CGTAGTGGCGAGCTGGAGTG
*VvrsbR* (VV2_0073)	Forward: CGATTGTCTGGTCTCGGGCG Reverse: TTCGAGCGCATCTCGCAAGG
*VvD1* (VV2_0077)	Forward: CATGCGGCACTGTTTCTGCG Reverse: CTTCCGCTTTCGCTTTGGCG
*pta* (VV1_2220)	Forward: CGCCACGAGACAGGTCGTTT Reverse: GGCAGCGTCTAAAGCGCCTA
*acsA* (VV1_1237)	Forward: CGCGCACCATCTCCGGTAAA Reverse: AGCCTGCACTGGTGGACAAC
*aceA* (VV1_0449)	Forward: ACGCATTCAGCACACCACCA Reverse: CAATTCGTTCCGTCGCGCAG
*aceB* (VV1_0450)	Forward: CATTCCCCGAGATCCACGCC Reverse: TCACTCGCCAAGACGATGCG
*glcB* (VV2_1647)	Forward: TGCTGGGTTGCCACAAAGGT Reverse: AAGGTCGTGCGCGTTTCCAA
*rpoS* (VV1_1588)	Forward: CCAGAGCGTGGTTTCCGCTT Reverse: GAGAAAGCTCACGCGCCGTA
*toxR* (VV1_0190)	Forward: ATGCTGGCACGTCAACAAAGATGG Reverse: TGGTGAGCAAGACAACGCAAAGTG
*fleQ* (VV1_1931)	Forward: TGGCGAACTTGGTTGAGCGT Reverse: TTCCAACACGTCGCGCTCTT

### 2.6 Acid stress survival assay

To test survival ability in CDM at pH 5, the three strains were grown in 2 mL LBN broth for a minimum of 17 h. Each culture was then washed and diluted to OD_600_= 0.1 in CDM adjusted with HCl to pH 5.0, in the absence of glucose, and incubated at 37 °C. To determine the number of viable cells, an aliquot was withdrawn at specific time points (0, 2, 4, 5, 6, 7, 8 and 24 h) and used to determine the number of CFU/mL, through ten-fold serial dilutions and colony counting on LBN agar. Three biological replicates were tested for each strain, each one plated in duplicate.

### 2.7 Motility assay

In order to determine swimming on CDM, the three strains were grown overnight in CDM broth. Plate counting was performed on overnight cultures as previously described to verify that all the strains were viable after overnight growth in CDM. A sterilized metal wire was dipped in the overnight culture and used to pierce the centre of the CDM motility plates (agar concentration 3.35 g/L). Plates were incubated at the appropriate temperature and the motility zone was measured after 16 h. Three biological replicates were tested for each strain.

### 2.8 Biofilm production

To assess biofilm formation in *V. vulnificus* CMCP6 and the two stressosome mutants, strains were grown in biological triplicates overnight in CDM broth. Plate counting was performed on overnight cultures to verify that all the strains were viable after growth in CDM. The overnight culture was then diluted in fresh CDM broth at a final OD_600_ of 0.1 and 200 µL cell suspension, assessed in three technical replicates, added to wells of a microtiter plate. The plates were incubated statically at 30 °C or 37°⁰C for 24 h and biofilm quantified through crystal violet staining (O'Toole et al., 1999). Briefly, the plate was washed twice with sterile PBS and dried at 60 °C for 1 h. 200 µL of a 0.1% (w/v) crystal violet solution were used to stain the biofilm and incubated at room temperature for 15 min. Plates were again washed with PBS three consecutive times and crystal violet dissolved using a 5% (v/v) acetic acid/water solution. OD_595_ was measured in a Sunrise microtiter plate, after transferring the resuspended crystal violet to a clean microtiter plate.

### 2.9 Cytotoxicity assay

To study cytotoxicity of *V. vulnificus* WT and stressosome mutant strains, HeLa cell lysis was assessed following co-incubation with bacterial cells using a protocol similar to that developed for *Vibrio parahaemolyticus* (Matlawska-Wasowska et al., 2010). HeLa cells were seeded in a 48-well plate with DMEM (Dulbecco's Modified Eagle Medium) + 10% fetal Bovine Serum (FBS) + 1% Pen/Strep as the growth medium. The HeLa cells were washed twice with DMEM + 10% FBS without antibiotics and a 30 min settling time allowed before adding the bacteria. Bacterial cultures were grown overnight in LBN broth or CDM. The overnight cultures in CDM were used directly for co-incubation. The overnight cultures in LBN were diluted in fresh LBN broth to a final OD_600_ of 0.05 and grown to mid-log phase (OD_600_ between 0.4 and 0.6). Bacterial cultures were first diluted to an OD_600_ of 0.01 in PBS and then added to the HeLa cells to achieve an MOI of 10. The plate was incubated at 37 °C and HeLa cells lysis was determined at different time points, through Lactate Dehydrogenase (LDH) quantification, using CytoTox 96 Non-Radioactive Cytotoxicity Assay (Promega), according to the manufacturer's protocol. HeLa cells treated with 1X Lysis buffer provided by the manufacturer were used as the positive control (100% lysis). HeLa cells in the absence of bacteria were used as the negative control (0% lysis). For each strain at least two biological replicates, each one assessed with technical triplicates, were analysed in at least two independent experiments.

### 2.10 Protease production

To test the production of protease, all strains were grown overnight in LBN broth and inoculated onto LBN plates containing 1% (w/v) skim milk clearing zones were measured after 24 h incubation at 37 °C.

## 3 Results

### 3.1 The stressosome is expressed in Fe-limited chemically defined media, in parallel with glucose depletion and acetic acid production

**Fig. 1:**
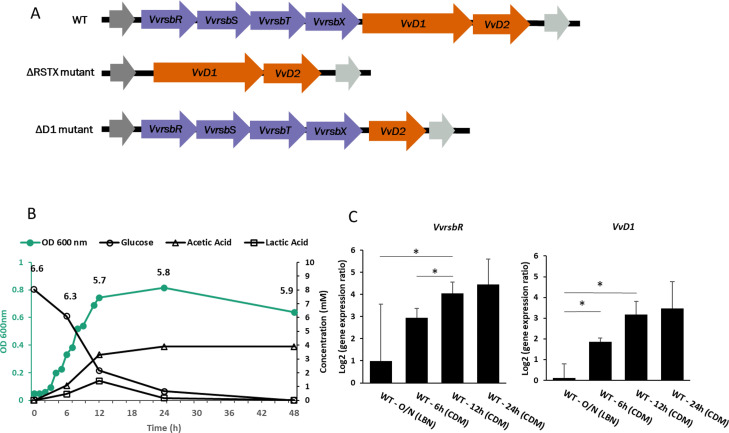
The stressosome locus in *Vibrio vulnificus*. A) The genetic stressosome locus organisation in *V. vulnificus* CMCP6 wild-type and in the two mutant strains (ΔRSTX and ΔD1). In purple the upstream module, made by *VvrsbR, VvrsbS, VvrsbT and VvrsbX* and in orange the downstream module made by *VvD1* and *VvD2*. Not drawn to scale. B) Growth curve of *V. vulnificus* CMP6 wild-type (green) in CDM + 0.15% Glucose + 0.75 µM FeCl_3_ at 37 °C with the concentrations of glucose (black circle), acetic acid (black triangle) and lactic acid (black squares) in the growth medium shown in black (HPLC quantification). The growth curves are the mean of the nine biological replicates, the HPLC reported values are the mean of three biological replicates. The numbers at the top of the graph represent the average pH of the media at 0, 6, 12, 24 and 48 h growth. C) *VvrsbR* and *VvD1* genes transcription in *V. vulnificus* CMCP6 wild-type after overnight growth in LBN and at 6, 12 and 24 h growth in CDM. The Log_2_ of the ratio between expression at each time point and expression in LBN O/N is represented. Reported values are the mean of the three biological replicates. Errors bars represent standard deviation. Student's *t*-test was performed comparing expression at different time points and p-values are shown (* <0.05).

The physiological role of the stressosome in *V. vulnificus* has so far not been characterised *in vivo*. To determine the conditions in which the stressosome may be physiologically active, transcriptional analysis of the upstream (*VvrsbR, VvrsbS, VvrsbT, VvrsbX*) and downstream (*VvD1, VvD2*) modules of the stressosome locus (Fig. 1A) in *V. vulnificus* CMCP6 was performed. Considering that the stressosome was expected to respond to stress conditions, we analysed transcription of *VvrsbR* and *VvD1* by qRT-PCR in bacteria during growth in an iron-limited chemically defined media (CDM), which represents periods of stress. Effects on *V. vulnificus* wild-type growth of different iron concentrations were investigated and the concentration of 0.75 µM was identified as growth-limiting and used in this work (Fig. S1). For the transcriptional analysis, bacteria were cultured for 16 h in LBN and, following the shift from nutrient-rich media (time point 0 h) to CDM, transcription was quantified at 6, 12 and 24 h (Fig. 1B, C). For both genes, transcription was detected in LBN and in CDM. In LBN expression levels were significantly lower than in CDM, where a significant upregulation was observed in late-log phase cells. The upregulation of the *VvrsbR* and *VvD1* gene in CDM is consistent with previous observations of stressosome expression in ASW (Williams et al., 2014) and CDM (Heinz et al., 2022). Based on these and previous observations, our hypothesis is that the stressosome is physiologically active in CDM, due to stimuli unique to these growth conditions.

To track changes in the media that could constitute the trigger for the stressosome expression at the late exponential phase in CDM, HPLC analysis of the media was performed to identify and quantify consumption of glucose, which was provided as a carbon source, and the secretion of overflow metabolites acetate and lactate, at 0, 6, 12, 24 and 48 h growth in CDM (Fig. 1B). Analysis of the media highlighted complete glucose depletion within 24 h, which correlates with the progressive increase of transcription of stressosome genes (Fig. 1C). This is in accordance with the previously reported starvation-dependant detection of the *Vv*RsbR stressosome protein and both *VvrsbR* and *VvD1* mRNA in the same organism (Heinz et al., 2022). At the same time, the accumulation of acetic acid (followed by a drop in pH from 6.6 to 5.7) was observed (Fig. 1B). This acetic acid production has been previously reported in several organisms including *Vibrio* spp., during growth in the presence of glucose (Ottaviani et al., 2001; Namdari and Cabelli, 1989). Moreover, an occasional and transient accumulation of lactic acid was observed after 12 h of growth.

These experiments confirmed that the stressosome in *V. vulnificus* is induced in CDM compared to a nutrient-rich media, such as LBN. Chemical characterisation of the media, during the growth of this organism, revealed the occurrence of various changes that could function as stimuli for the activation of the stressosome in *V. vulnificus*. Amongst these, primarily glucose depletion and acetic acid accumulation caused by glucose metabolism, and iron-limitation due to the media itself. These potential stimuli will be investigated further in this work to explore possible physiological roles of the stressosome in *V. vulnificus*.

### 3.2 Knockout mutation of the stressosome genes has no detectable physiological effect on *V. vulnificus* in nutrient-rich media

In order to investigate the *in vivo* role of the stressosome in *V. vulnificus*, two knockout stressosome mutants were constructed using *V. vulnificus* CMCP6 as the parental strain, the ΔRSTX mutant lacking the whole upstream module and the ΔD1 lacking the sensor kinase of the downstream two-component regulatory system (Fig. 1A). The mutations were confirmed through PCR (Fig. S2) and whole-genome sequencing (WGS) analysis was performed for the wild-type (WT) strain and the two stressosome mutants. The sequencing results were aligned and analysed using *V. vulnificus* CMCP6 chromosome I and II sequences (GenBank: AE016795.3 and AE016796.2) as reference sequences and by comparing the mutants with the wild-type. The WGS confirmed the knockout mutations and excluded the presence of secondary mutations shared between the two mutants or other mutations that could explain the phenotypes presented in this work (data available upon request). Growth characterisation of both mutants in LBN showed no differences in growth rate and overall biomass accumulation between these and the wild-type strain (Fig. S3). Moreover, an extensive phenotypical characterisation of the ΔRSTX mutant in LBN showed no differences caused by the mutation. The mutant was tested for growth, survival and tolerance to several stresses, cross-protection and motility in rich media and showed physiological traits identical to the wild-type strain (Cutugno et al., 2022). These results, together with the previously discussed transcriptional analysis of the stressosome locus, indicate that in *V. vulnificus* the stressosome is most likely active in a Fe- and nutrient-limited environment.

### 3.3 The downstream TCS is required for up-regulation of the glyoxylate shunt pathway during growth in CDM

As shown above, the stressosome is upregulated during growth in CDM, when glucose is depleted and acetic acid produced. For this reason, phenotypic characterisation of the ΔRSTX and ΔD1 strains was performed in CDM in this study. The effects of each mutation on the transcription of the genes in the other module were tested through qRT-PCR.Deletion of the upstream module seemed to reduce transcription of the downstream *VvD1* gene, while transcription levels of *VvrsbR* were similar in CMCP6 and ΔD1 (Fig. S4).

**Fig. 2:**
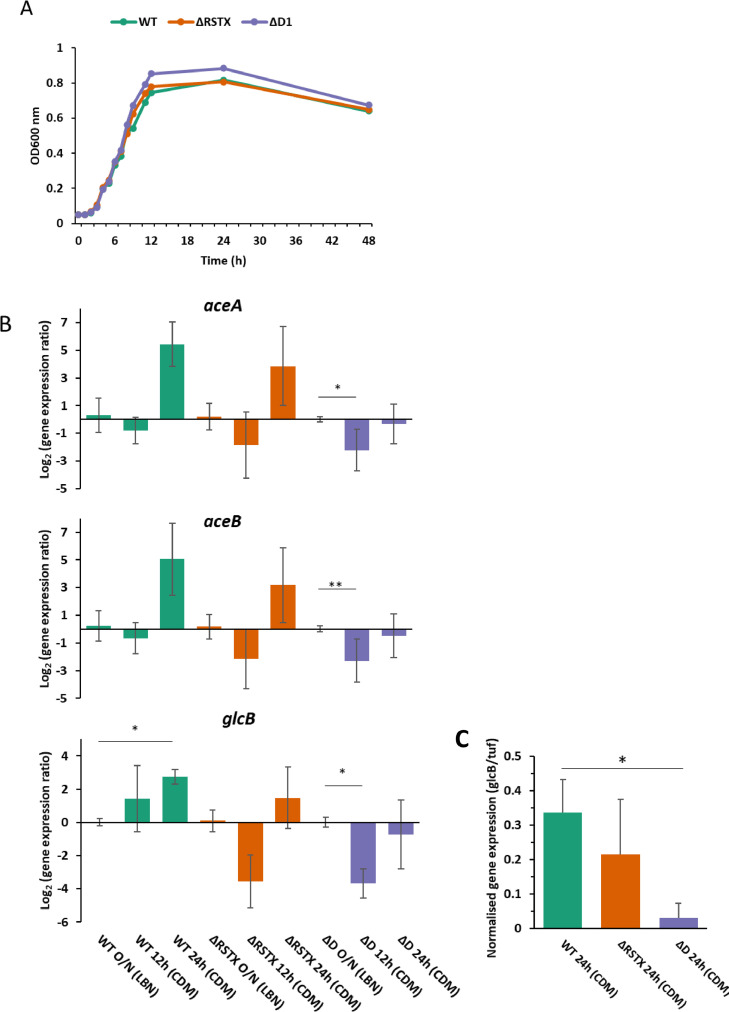
Growth and metabolism of the stressosome mutants in CDM. A) Growth curve of *V. vulnificus* CMP6 wild-type (green), ΔRSTX (orange) and ΔD1 (purple) in CDM + 0.15% Glucose + 0.75 µM FeCl_3_ at 37 °C. The curves are the mean of nine biological replicates. B) *aceA, aceB* and *glcB* gene transcription in *V. vulnificus* CMCP6 wild-type and stressosome mutants after overnight growth in LBN and at 12 and 24 h growth in CDM. The Log_2_ of the ratio between expression at each time point and expression in LBN O/N is represented. Reported values are the mean of three biological replicates. C) *glcB* gene transcription in *V. vulnificus* CMCP6 wild-type and stressosome mutants after 24 h growth in CDM. The normalised gene expression level is represented. Reported values are the mean of three biological replicates. Student's *t*-test was performed comparing expression at different time points and p-values are shown (*<0.05; ** <0.01).

We first investigated the growth and metabolic activity of the ΔRSTX and ΔD1 strains of *V. vulnificus* in CDM*.* The growth measurements revealed a partial growth advantage of ΔD1 in the late-log and early-stationary phase (Fig. 2A), which corresponds to the time when glucose depletion occurs and the stressosome genes are upregulated. The HPLC analysis on the culture media of the two mutants demonstrated that the kinetics of glucose consumption and acetic acid secretion were similar in the wild-type and the two mutant strains (Fig. S5). Also, the same transient accumulation of lactic acid at 12 h growth was observed (Fig. S5). pH changes of the media during growth were similar for both wild-type and mutants (Fig. S5). This excludes differential glucose consumption rate or differential acid production rate as a possible explanation for the growth difference. It instead suggested that the stressosome might alter the response of *V. vulnificus* to changes in the culture medium during growth.

The next step was to test, through quantitative RT-PCR (qRT-PCR), whether metabolic pathway alternatives to the glycolysis/TCA cycle were differentially regulated in the mutants when compared to the wild-type during growth in CDM. Several intermediates of the glucose central metabolic pathway can be utilised for alternative metabolic pathways, such as acetate metabolism, glyoxylate shunt and gluconeogenesis, based on various environmental factors including oxygen levels, iron availability and nature and quantity of the carbon source (Wolfe, 2005). As previously described, the ΔD1 strain showed an increased OD during growth in CDM, however, no difference in glucose consumption and acetate secretion between strains was observed. This led to the hypothesis that the glycolysis step and *pta*-dependant acetate production/secretion should be unchanged*.* To test this hypothesis, the transcription of the *pta* and *acs* genes was investigated through qRT-PCR. These two genes encode a phosphotransacetylase involved in the acetate dissimilation pathway and an acetyl-CoA synthase involved in the acetate assimilation pathway, respectively, and have been widely studied as part of the acetate switch in bacteria, including *Vibrio* spp. The acetate switch occurs when bacteria switch from a metabolic pathway that utilises acetogenic carbon sources (e. g. d-glucose) and accumulates acetate, to a pathway, generally characterised by a lower growth rate, that facilitates the utilisation of that acetate that was previously accumulated. The acetate switch has been widely studied and can be considered as a survival pathway that occurs when the principal carbon source has been depleted (Wolfe, 2005; Kim et al., 2015; Studer et al., 2008). In this work, no significant difference was observed in the transcriptional levels or the regulation pattern of these genes (Fig. S6). This, together with the similar profiles of acetic acid accumulation, exclude an effect of the stressosome on the utilisation of acetyl-CoA in the acetate metabolism.

One of the previously hypothesised roles of the stressosome in *V. vulnificus* is that it functions as an iron-sensing complex (Heinz et al., 2022). Moreover, since acetyl-CoA can be used either by the TCA cycle or the iron-regulated glyoxylate shunt, resulting in different energy yields and consequently growth, the balance between both pathways could be the reason for the observed growth difference. To test this hypothesis, the regulation of genes involved in the glyoxylate shunt was investigated. The glyoxylate shunt functions as an alternative to the TCA cycle and allows acclimation to low iron concentration in marine gammaproteobacteria and other bacterial species (Koedooder et al., 2018; Ha et al., 2018). This metabolic pathway is advantageous in iron-limited growth conditions because it redirects the bacterial metabolism away from the electron transport chain and its Fe-dependant enzymes and allows the conservation of atoms of carbon (normally utilised to produce CO_2_ in the TCA cycle), through the production of oxaloacetate and subsequent gluconeogenesis (Koedooder et al., 2018; Tortell et al., 1996; Kornberg and Krebs, 1957). The transcription of the two genes involved in the glyoxylate shunt, *aceA* (encoding isocitrate lyase) and *aceB* (encoding malate synthase), was tested together with the *glcB* gene encoding a malate synthase-related protein (Fig. 2B and 2C). qRT-PCR data revealed that the WT up-regulates these genes in stationary phase at 24 h growth in CDM, while the downstream ΔD1 strain not only does not up-regulate this pathway during growth in CDM but even significantly down-regulates it at the 12 h time-point. In the ΔRSTX strain, the regulation of the glyoxylate shunt was intermediate between the wild-type and the ΔD1 strain. We observed an initial down-regulation of the pathway (in particular of the *glcB* gene) following the shift from LBN to CDM, similar to the one observed in the ΔD1 strain. Each of the analysed genes was upregulated at subsequent time points although with lower transcription levels than the wild-type. Thus during the stationary growth phase, gene transcription in the ΔRSTX strain followed a pattern similar to that of the wild-type.

### 3.4 The stressosome mutants survive acid stress, through a pathway that is *rpoS*- and *toxR*-independent

As described above, *V. vulnificus* accumulates acetic acid and lactic acid during growth in CDM in the presence of glucose. The accumulation of organic acids causes a significant pH drop in our experimental conditions and this has been previously reported to cause loss of viability in several bacterial species through the so-called “suicide phenomenon” (Namdari and Cabelli, 1989). To investigate whether the stressosome plays a role in the response of *V. vulnificus* to acidity and whether this could contribute to the extended growth profiles of the ΔD1 mutant, viability was quantified by plate counts during growth in CDM.

**Fig. 3:**
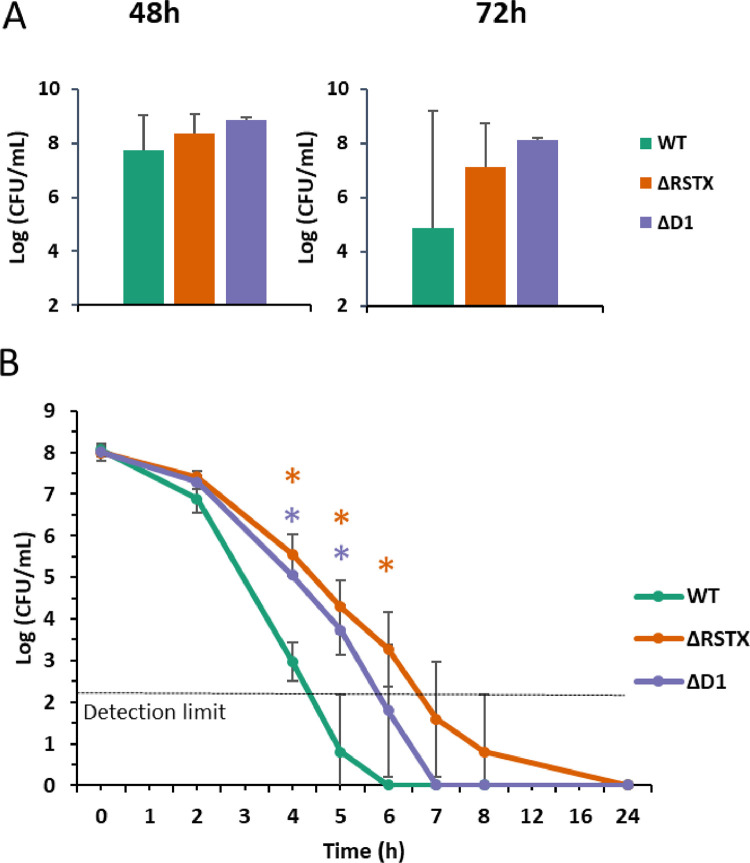
Acid stress resistance. A) Cell viability of *V. vulnificus* wild-type (green), ΔRSTX (orange) and ΔD1 (purple) was assessed, though plate counting, after 48 and 72 h growth in CDM + 0.15% Glucose + 0.75 µM FeCl_3_ at 37 °C. For each time point, the number of viable cells was plotted as Log_10_(CFU/mL). Three biological replicates for each strain were tested and the reported values are the mean of the three replicates. B) Survival assay in CDM at pH 5 of *V. vulnificus* wild-type (green), ΔRSTX (orange) and ΔD1 (purple). The strains were incubated at 37 °C in CDM at pH 5 and survival was assessed, through plate counting, at eight different time points (0, 2, 4, 5, 6, 7, 8 and 24 h). For each time point, survival was plotted as Log_10_(CFU/mL). Three biological replicates for each strain were tested and the reported values are the mean of the three replicates. Errors bars represent standard deviation. Student's *t*-test was performed comparing mutant strains to WT and p-values are shown (* <0.05). Detection limit represents the minimum number of countable CFU, according to the described protocol.

Although a degree of variability was observed, the viability of the wild-type and mutants was similar after 48 h growth (OD_600_= 0.6 and pH 5.9), so the extended growth profile was not due to alterations in the bacterial response to stress. However, after 72 h growth, the viability of the wild-type strain decreased more than the mutants (Fig. 3A). This revealed an average 1000-fold decrease in viability of the wild-type from 10^8^ CFU/mL at 48 h to 10^5^ CFU/mL after 72 h growth and only a 10-fold decrease in viability of the two mutant strains between 48 and 72 h of growth. Large differences in the viability of the wild-type strain caused this difference to be not statistically significant but showed a unique viability pattern that could have an interesting biological meaning. Dead/Live staining was performed on the wild-type to determine whether the cells were dead or in a Viable But Non-Culturable (VBNC) state. The results indicated that they were indeed dead as indicated by loss of membrane integrity in more than 95% of CMCP6 cells after 72 h growth, as determined by cellular influx of membrane-impermeable fluorescent dye. .

To explore the possibility of a differential acid stress response, the three strains were tested for acid survival in CDM pre-acidified to pH 5 with HCl in the absence of glucose, to mimic the combination of starvation and acid stress at the end of the growth curve. Both mutants showed a significantly longer survival at low pH when compared to the WT (Fig. 3B). The wild-type viability was below the detection limit already after 5 h of exposure to pH 5, while the ΔD1 and the ΔRSTX strains were still viable after 5 and 6 h of exposure, respectively. This was in line with the results of the plate count at the end of the growth curve.

**Fig. 4:**
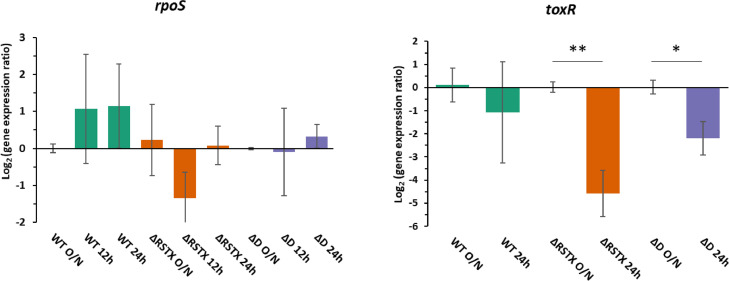
Global stress regulators transcription. *rpoS* and *toxR* transcription in *V. vulnificus* CMCP6 wild-type and stressosome mutants after overnight growth in LBN and at 12 h and/or 24 h growth in CDM. The Log_2_ of the ratio between expression at each time point and expression in LBN O/N is represented. Reported values are the mean of the three biological replicates. Errors bars represent standard deviation. Student's *t*-test was performed comparing expression at different time points and P values are shown (* <0.05; ** <0.01).

In order to elucidate the molecular mechanism underlying this surprising phenotype, the transcription of the two global stress regulators *rpoS* and *toxR* was studied in the wild-type and the mutants after overnight growth in LBN and at different time points in CDM (Fig. 4). Both *rpoS* and *toxR* are involved in acid stress response in several *Vibrio* spp., including *V. vulnificus* (Hülsmann et al., 2003; Chen et al., 2010; Merrell et al., 2001). The qRT-PCR showed the absence of any significant regulation of *rpoS* in our experimental conditions for the wild-type and no significant differences between the wild-type and the mutants. Concerning *toxR*, we observed a significant downregulation of this gene after 24 h growth in CDM in both mutants, while a lesser reduction occurred in the WT. Neither of these results explains the acid survival advantage of the stressosome mutants and it suggests that such advantage is due to the utilisation of a *rpoS-* and *toxR-*independent pathway. Further investigation will be essential not only to elucidate the advantage observed in the absence of the stressosome but also to explore alternative pathways for acid survival in *V. vulnificus*.

### 3.5 The stressosome confers a significant advantage in motility on CDM motility plates

The results shown so far demonstrate that the presence of the stressosome represents a significant disadvantage in both growth and the stress survival phenotypes investigated. Although in contrast with the previously identified roles of the stressosome in Gram-positive bacteria (Chen et al., 2003), these data raise an interesting question about the selective advantage of such a complex in *V. vulnificus*. The putative role of the downstream module in regulating the intracellular levels of cyclic-di-GMP (Pané-Farré et al., 2005) and the observed downregulation of *toxR* in the stressosome mutants, suggests a role of the stressosome in the regulation of infection-related traits, such as motility, biofilm formation and cytotoxicity (Chen et al., 2018).

**Fig. 5:**
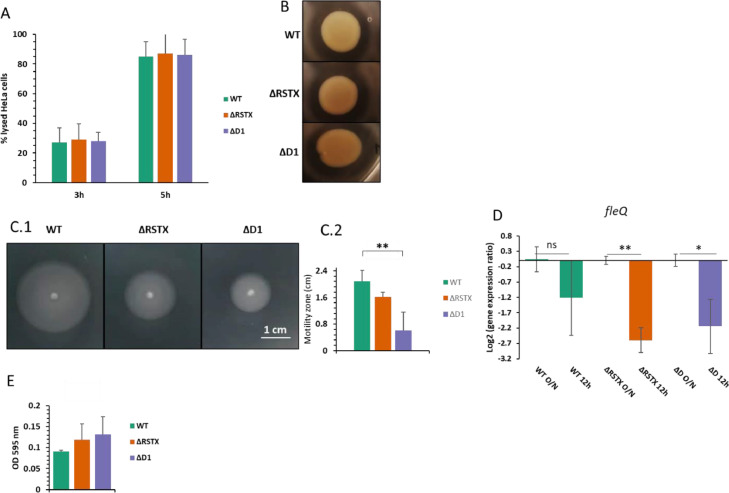
Motility and virulence characterisation. A) Cytotoxicity assay, co-incubation experiment with HeLa cells and LDH quantification. Percentage of lysed HeLa cells, compared to a 100% lysis control, was calculated through LDH quantification at different time points after co-incubation with *V. vulnificus* CMCP6 wild-type and the stressosome mutants. At least two biological replicates for each strain were tested and the reported values are the mean of the replicates. Errors bars represent standard deviation. B) Protease production was tested on LBN agar plates + 1% skimmed milk. Pictures were taken after 24 h incubation at 37 °C. C) Motility assay on CDM motility plates of *V. vulnificus* wild-type (green), ΔRSTX (orange) and ΔD1 (purple). All strains were stabbed on motility CDM agar plates and incubated at 37 °C. Pictures of the motility plates were taken after 16 h incubation (C.1) and the motility zone was measured (C.2). Three biological triplicates for each strain were tested and the reported values are the mean of the three replicates. Errors bars represent standard deviation. Student's *t*-test was performed and p-values are shown (**<0.01). D) *fleQ* gene transcription in *V. vulnificus* CMCP6 wild-type and stressosome mutants after overnight growth in LBN and at 12 h growth in CDM. The Log_2_ of the ratio between transcription at 12 h and transcription in LBN O/N is represented. Reported values are the mean of the three biological replicates. Errors bars represent standard deviation. Student's *t*-test was performed comparing transcription at different time points and P values are shown (* <0.05; ** <0.01). E) Biofilm accumulation assay in CDM + 0.15% Glucose + 0.75 µM FeCl_3_ at 37 °C. Biofilm was quantified after 48 h growth and OD_595_ was measured following crystal violet staining. At least three biological replicates for each strain were tested and the reported values are the mean of the replicates. Errors bars represent standard deviation.

To test the possibility of stressosome-mediated virulence regulation in *V. vulnificus,* the effects of the stressosome mutations on the virulence of *V. vulnificus* were investigated, by evaluating cytotoxicity effects on HeLa cells and protease production (Fig. 5A and 5B). Cytotoxicity effects of the wild type and the ΔRSTX and the ΔD1 strains of *V. vulnificus* on HeLa cells were evaluated in terms of cell lysis, through LDH quantification in comparison to a positive lysis control. All three strains were grown to mid-log phase in LBN (Fig. 5A) or stationary phase in CDM (data not shown) and co-incubated with HeLa cells. Extracellular LDH was quantified at different time points following co-incubation and showed that the three strains induced similar lysis rates in the eukaryotic cells and caused more than 80% lysis within 5 h co-incubation in the case of exponentially growing bacteria pre-cultured in LBN. It took longer for bacteria pre-cultured in CDM to cause cell death than those pre-cultured in LBN (less than 10% cytotoxicity detected for each strain at 5 h co-incubation), though ultimately equal levels of cytotoxicity were detected for each strain after 8 h co-incubation (95, 99 and 97% HeLa cell lysis for WT, ΔRSTX and ΔD1, respectively). Protease production was tested by spotting overnight bacterial cultures on LBN + 1% skimmed milk agar, zones of clearing were measured following 24 h incubation (Fig. 5B). Protease production could not be evaluated on CDM because the addition of skimmed milk to CDM agar plates caused significant precipitation in the media. The results of these experiments showed that, despite the differential regulation of *toxR,* the stressosome does not influence cytotoxicity or extracellular protease production. Due to the putative role of the stressosome in regulating c-di-GMP and the well-known correlation between motility and virulence in *Vibrio* spp. (Kim et al., 2014; Ran Kim and Haeng Rhee, 2003; Guentzel and Berry, 1975; Skorupski and Taylor, 1997), motility assays were performed on CDM motility agar (Fig. 5C). The wild-type showed a motility zone more than double the diameter of the downstream mutant, indicating a clear motility defect due to the TCS system deletion. Moreover, both mutants significantly downregulated the transcription of the motility regulator gene *fleQ* after 12 h growth in CDM, while a lesser reduction occurred in the wild type (Fig. 5D). While the strongest repression of *fleQ* is in the △RSTX strain and the strongest inhibition of motility is for the ΔD1 strain, the reductions in these characteristics are not significantly different between the two strains. Flagellar synthesis in bacteria follows a hierarchical cascade and *fleQ* (that shares more than 80% homology with *flrA* in *V. cholerae* and *flaK* in *V. parahaemolyticus*) was first identified in *Pseudomonas aeruginosa* as the master gene at the top of the hierarchy (Jyot et al., 2002; Khan et al., 2020; Echazarreta and Klose, 2019; Kim and McCarter, 2000). FleQ regulates the expression of all the other flagellar genes by binding the sigma factor σ^54^ and it is regulated by c-di-GMP (Khan et al., 2020; Jenal et al., 2017; Prouty et al., 2001), which could explain the observed effects of the studied mutations and in particular the deletion of the downstream two-component regulatory system. Downregulation of this gene in the stressosome mutants explains the decreased motility and encourages further investigation to better elucidate the link between the stressosome and the regulation of *fleQ*.

Motility and biofilm formation are inversely regulated by cyclic-di-GMP (Jenal et al., 2017). Accordingly inactivation of the stressosome may lead to increased biofilm production. For this reason, biofilm formation in CDM was tested, through crystal violet staining (Fig. 5E). Biofilm formation by wild type and mutant *V. vulnificus* was only minimally detected in standard biofilm growth conditions. Despite assessing a variety of different growth conditions, biofilm production was only marginally increased, if at all, by either the WT or the stressosome mutants. Conditions amended were growth temperature, growth media, culture time, glass and polystyrene substrates, glucose and NaCl concentrations and size of inoculum. Our data show that the stressosome mutations did not substantially increase biofilm formation.

## 4 Discussion

The stressosome is a large molecular complex that works as a signalling hub in different bacterial species, regulating stress response in the environment and during infection of pathogenic bacteria (Chen et al., 2003; Kim et al., 2004; Marles-Wright et al., 2008). The stressosome was primarily characterised in Gram-positive bacteria such as *B. subtilis* and L. *monocytogenes* where, following certain environmental stimuli, it activates the alternative sigma factor σ^B^ (Yang et al., 1996; Gaidenko et al., 1999; Eymann et al., 2011) with subsequent expression of a large number of genes involved in stress response (Guerreiro et al., 2020; Peterson and Mekalanos, 1988). The stressosome genes were identified in several bacterial species, including numerous Gram-negatives, belonging to several phyla, and bacteria that do not possess σ^B^, and amongst these is the human pathogen *V. vulnificus* (Pané-Farré et al., 2005). This characterisation of the *in vivo* role of the stressosome in *V. vulnificus* is the first in a Gram-negative organism and it is particularly significant to identify the regulatory outputs of a stressosome that likely acts independently of a downstream sigma factor. Previous *in vitro* studies have suggested possible roles for the stressosomes of *V. brasiliensis* and *V. vulnificus* in oxygen-sensing and iron metabolism (Jia et al., 2016; Heinz et al., 2022).

In this work, the construction of two stressosome mutants followed by phenotypical and transcriptional characterisation has shed light on the possible functions of this complex in *V. vulnificus*. The mutagenesis strategy targeted both the upstream module of the stressosome locus, containing the genes forming the complex (*VvrsbR, VvrsbS, VvrsbT, VvrsbX*), and part of the downstream module formed by two genes (*VvD1* and *VvD2*) encoding a two-component regulatory system most likely involved in the degradation of c-di-GMP and hypothesised to be the regulatory output of the stressosome in *V. vulnificus* (Pané-Farré et al., 2005; Heinz et al., 2022). Transcription profiling of the stressosome genes in the CMCP6 wild-type strain during growth in CDM has revealed that the stressosome genes are significantly upregulated in CDM compared to LBN and that this upregulation coincides with glucose depletion and acetic acid production. A similar pattern has been observed at the mRNA and protein level (Heinz et al., 2022) and it indicates that the stressosome is physiologically active in nutrient-limited growth conditions. To investigate the role of the stressosome in rich media, an extensive phenotypical characterisation of the upstream mutant (ΔRSTX) has been previously carried out in LBN and no significant differences were observed in growth, stress response, motility or virulence (Cutugno et al., 2022). As a consequence, most of the subsequent phenotypic characterisation reported in this work was performed in CDM.

We first focused on the growth and metabolism of *V. vulnificus*. During growth in CDM, the downstream mutant (ΔD1) show a partial growth advantage in the late exponential phase and early stationary phase, at the time in which the transcription of the stressosome genes reaches its peak in the wild-type strain. Despite the higher growth, no differences in the rate of consumption of glucose or accumulation of organic acids were observed. To investigate the possible causes of this growth difference, the transcription of the genes involved in metabolic pathways alternative to the TCA cycle was tested. In particular, we targeted the acetate metabolism pathway (Wolfe, 2005) and the glyoxylate shunt (Kornberg, 1966), the first one because of the accumulation of acetic acid during growth in CDM and the second because of its well-established role in acclimation to iron-limitation (Koedooder et al., 2018). We demonstrated that the wild-type and the ΔD1 strain differentially regulate the genes involved in the glyoxylate shunt. The wild-type upregulates *aceA, aceB* and *glcB* in stationary phase, after 24 h growth in CDM, as expected in iron-limited conditions and as previously demonstrated in other organisms (Koedooder et al., 2018; Ha et al., 2018). In contrast, the ΔD1 strain does not upregulate these genes during growth in CDM and it even downregulates them in the early stage of growth. This difference in the regulation of the glyoxylate shunt could explain the growth advantage of the ΔD1 strain at the late exponential and early stationary phase because this pathway, although useful for coping with iron-limitation, cannot replace the ATP generated by the TCA cycle (Koedooder et al., 2018; Kornberg, 1966; Ha et al., 2018; Kornberg and Krebs, 1957). When the iron concentration is low and the activity of TCA cycle enzymes is compromised by the limitation of the electron transport chain, activation of the glyoxylate shunt pathway might occur via the stressosome. While this diverts substrates away from the TCA cycle enabling continuing carbon metabolism and essential metabolite production, it could be energetically disadvantageous compared to the TCA cycle and thereby translate into reduced growth for the wild-type. The absence of the downstream two-component system prevents the activation of the glyoxylate shunt and promotes continued, though compromised, utilisation of the classical TCA cycle, which provides an extended period of growth and a delayed entry into stationary phase by the ΔD1 strain. This could be caused by the inability of the stressosome TCS mutant to sense and respond to iron limitation and subsequent lack of activation of the glyoxylate shunt. Another possibility is that the up-regulation of iron-uptake molecules in the mutants would allow them to cope with iron depletion without having to redirect their metabolism toward the glyoxylate shunt. Previously published proteomic data have shown a general down-regulation of iron-uptake molecules in the ΔRSTX strain compared to the wild-type (Heinz et al., 2022). This is contradictory to the latter scenario and would rather align with the first scenario in which the stressosome mutants are less able to sense iron and subsequently less able to activate alternative non-Fe requiring metabolic pathways, such as the glyoxylate shunt. It is interesting to notice that, in our experimental conditions, the ΔRSTX strain showed a lesser growth advantage than the ΔD1 strain and an intermediate regulation profile of the *aceA, aceB* and *glcB* genes, with an initial downregulation similar to the one of the ΔD1 strain and a subsequent upregulation similar to the one of the wild-type. We hypothesise that the TCS retains some functionality in the ΔRSTX strain, possibly through cross-talk with other TCS systems or alternative activation of VvD1 via its PAS domain, resulting in intermediate phenotypes for this mutant and its similarity with the wild-type regarding growth and glyoxylate shunt expression.

In addition to the effects of these mutations on the growth and metabolism of *V. vulnificus*, we tested the effect on stress response and in particular acid stress survival. As previously reported (Ottaviani et al., 2001; Namdari and Cabelli, 1989) and as confirmed in this work, *V. vulnificus* accumulates acetic acid when growing in the presence of glucose. This causes progressive acidification of the media and loss of viability. This viability loss was observed in our experimental conditions and it seemed to affect the wild-type strain more than the two mutants. This difference is probably caused by a higher survival rate of the mutants which was further suggested by exposing the three strains to a pre-acidified CDM, in the absence of glucose. In line with the viability at the end of the growth curve, both mutants showed a higher survival to acid stress when compared to the wild-type.

Transcriptional analysis of the two global stress regulators *rpoS* and *toxR* revealed that *rpoS* was not significantly affected by the stressosome mutations in our experimental conditions and *toxR* was heavily downregulated in both mutants during growth in CDM. These results were not intuitive when compared to the phenotypic data and, although further investigation is needed, we speculate that the higher acid survival rate in the mutants is due to the activation of an alternative *rpoS-* and *toxR-*independent pathway, in the absence of the stressosome complex. Our observations so far, demonstrated that the presence of the stressosome confers a disadvantage both in growth and stress survival in *V. vulnificus*.

Due to the presence of this complex in a large proportion of *V. vulnificus* strains we investigated the possibility of a stressosome-conferred advantage in infection and virulence factors, such as motility, biofilm formation, cytotoxicity and protease production. Although no differences were observed between the wild-type and the mutants in terms of cytotoxic effects on HeLa cells or exoenzyme production, we demonstrated that the stressosome confers a significant advantage in motility. Moreover, the lack of the stressosome or its downstream module causes a significant downregulation of the motility regulator FleQ, probably at the base of such phenotypical difference. FleQ homologues in *V. cholerae* and *V. parahaemolyticus* are known to be regulated by c-di-GMP (Khan et al., 2020). This result fits with the hypothesis of a downstream module involved in c-di-GMP degradation (Pané-Farré et al., 2017; Pané-Farré et al., 2005) as this second messenger is a well-known motility repressor (Jenal et al., 2017) and higher c-di-GMP, due to the inactivity of the predicted phosphodiesterase in the downstream module, could be the cause of reduced motility in the mutant.

Global expression analysis comparing proteomes of *V. vulnificus* CMCP6 and ΔRSTX has previously been published (Heinz et al., 2022). We compared our qRT-PCR transcription analysis results with these proteomic analyses of bacteria grown in CDM in aerobic conditions. Both approaches demonstrated that *VvD1* (VV2_0077) was down-regulated in ΔRSTX compared to the wildtype. Both approaches also demonstrated that *acs* (VV1_1237), *pta* (VV1_2220) and *rpoS* (VV1_1588) were expressed at similar levels in WT and ΔRSTX. While *toxR* (VV1_0190) and *fleQ* (VV1_1931) transcription was down-regulated in ΔRSTX, there was no change in protein levels compared to the WT, possibly due to continued protein stability despite reduced de novo synthesis. qRT-PCR studies demonstrated an intermediate effect of deleting RSTX on *aceA* (VV1_0449), *aceB* (VV1_0450) and *glcB* (VV2_1647) transcription compared to ∆D1 and WT. Likewise there was minimal influence of RSTX on AceA or AceB protein levels, though GlcB levels did decrease.

These observations together represent the first *in vivo* characterisation of the stressosome in *V. vulnificus* and in a Gram-negative bacterium. This work provides the first insight into the effects that this locus can have on the physiology of this human pathogen. In line with the observation of an iron-dependant stressosome previously published (Heinz et al., 2022), we observed a growth advantage of the TCS mutant in a nutrient- and iron-limited growth condition. This is most likely due to the total or partial inability to activate the glyoxylate shunt in the absence of the stressosome downstream sensor kinase. In addition to that, we demonstrated that the stressosome is important for the motility of *V. vulnificus*, most likely through regulation of the intracellular levels of cyclic-di-GMP, and to maintain higher *toxR* transcription levels in the passage between rich and chemically defined media. These conditions may mimic those that *V. vulnificus* encounter upon entry into the gastrointestinal tract where there is limited iron available to the bacteria and also possibly limited availability of nutrients that *V. vulnificus* can readily metabolise, These results suggest that the stressosome in *V. vulnificus* could be important for the early stage of the infection process, during which iron-sensing and motility are essential factors for successful colonisation of *V. vulnificus* (Wright et al., 1981; Bullen et al., 1991; Kim et al., 2014; Ran Kim and Haeng Rhee, 2003; Guentzel and Berry, 1975). The similarity between the phenotypic effects of the two mutations allows us to speculate that they are functionally related, although the intermediate phenotypes showed by the upstream mutant when compared to the downstream one could indicate an alternate route for the regulation of the two-component regulatory system in the absence of the stressosome.

This first *in vivo* analysis of the stressosome lays the basis for the understanding of the function and regulatory hub mediated by the stressosome in *V. vulnificus* and it confirms the deep difference between the widely-characterised Gram-positive stressosome and the same complex in Gram-negative bacteria.

## FUNDING

This research was jointly supported by a Marie Skłodowska-Curie ITN Fellowship (PATHSENSE, project number 721,456) within the seventh European Community Framework Program and by the Irish Research Council (IRC), under grant number GOIPG/2020/1420. Additional support was provided by the Irish HEA (Higher Education Authority) as part of a COVID-19 costed extension programme.

## CRediT authorship contribution statement

**Laura Cutugno:** Conceptualization, Methodology, Investigation, Data curation, Validation, Formal analysis, Visualization, Writing – original draft, Funding acquisition. **Borja Khatabi Soliman Tamayo:** Methodology, Investigation, Formal analysis, Writing – review & editing. **Piet N.L. Lens:** Resources, Validation, Supervision, Writing – review & editing. **Conor O'Byrne:** Conceptualization, Methodology, Validation, Writing – review & editing, Funding acquisition. **Jan Pané-Farré:** Conceptualization, Methodology, Validation, Writing – review & editing, Supervision, Funding acquisition. **Aoife Boyd:** Conceptualization, Methodology, Validation, Resources, Data curation, Writing – review & editing, Supervision, Project administration, Funding acquisition.

## Declaration of Competing Interest

The authors declare that they have no known competing financial interests or personal relationships that could have appeared to influence the work reported in this paper.

## Data Availability

Data will be made available on request.

## Appendix Supplementary materials

Image, application 1
