# Supplementary material for: In vivo characterisation of the Vibrio vulnificus stressosome: A complex involved in reshaping glucose metabolism and motility regulation, in nutrient- and iron-limited growth conditions
Source: Curr Res Microb Sci. 2023 Mar 2;4:100186. doi: 10.1016/j.crmicr.2023.100186 (PMC10014275; doi:10.1016/j.crmicr.2023.100186)
Supplement: Supplementary file 1 [file mmc1.docx]

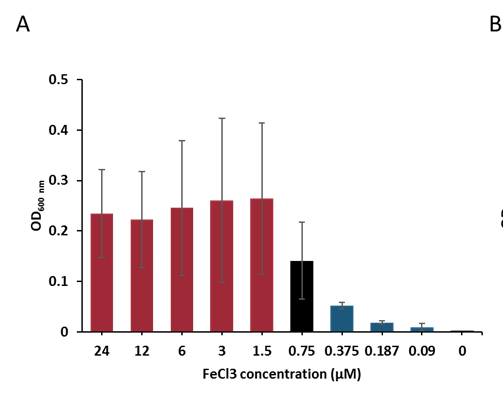

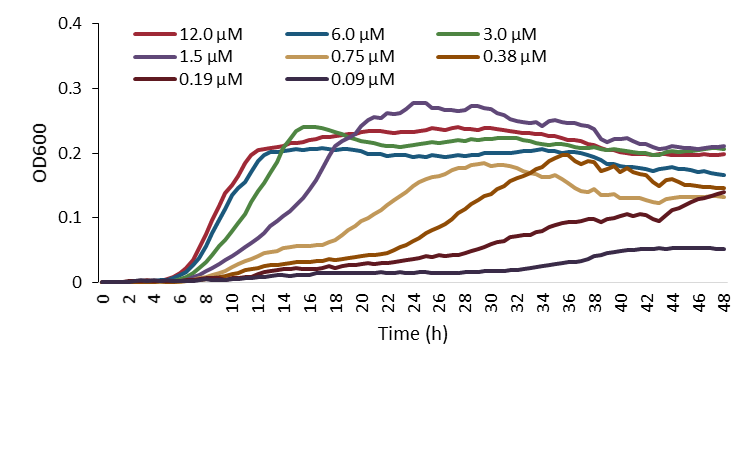


**Figure S1**. Effect of FeCl_3_ concentration on growth of *V. vulnificus* CMCP6 wild-type in CDM + 0.15% Glucose at 37°C. A) Minimum FeCl_3_ concentration required for growth. OD_600_ was measured after 24 h incubation in CDM broth supplemented with the indicated FeCl_3_ concentrations. The reported values are the mean +/- standard deviation of three biological replicates. Black corresponds to the iron concentration used throughout this study (0.75 µM), higher concentrations higher are shown in red (24 – 1.5 µM) and lower concentrations are shown in blue (0.375 – 0 µM). B) Growth curve in microtitre plates in CDM broth supplemented with the indicated FeCl_3_ concentrations. OD_600_ was measured every 15 min for 48 h. The curves are the mean of three biological replicates. For clarity error bars are omitted.


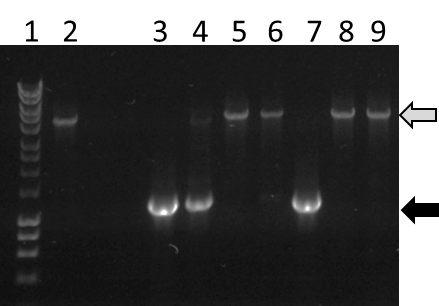


**Figure S2.** Confirmation of *V. vulnificus* ∆D1 construction by PCR. Colony PCR with D1_Rev and D1_For primers which anneal external to the deletion site was performed on *V. vulnificus* second recombinants. PCR products were separated by agarose gel electrophoresis and visualised by SYBR green fluorescent staining. Lane 1: DNA ladder, Lane 2: WT allele control (grey arrow indicates PCR product of 4 kb), Lanes 3-9: second recombinants. The 1 kb PCR products (indicated by black arrow) present in lanes 3,4 and 7 show that these *V. vulnificus* possess the ∆D1 allele.


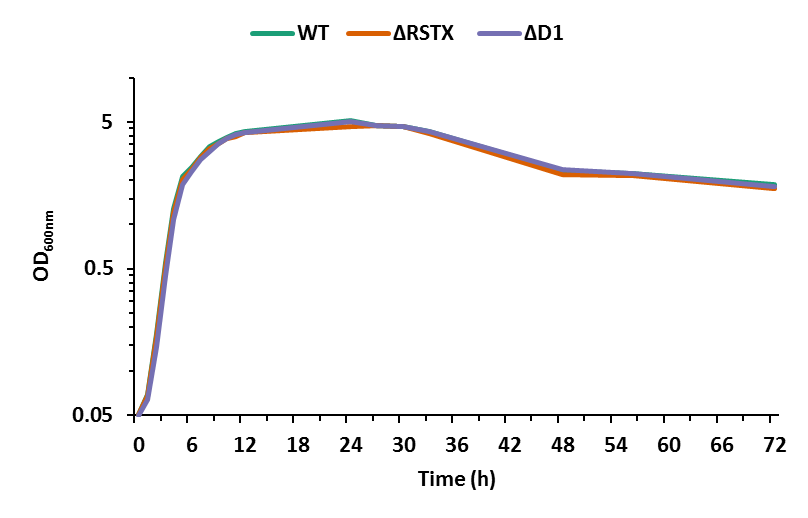


**Figure S3**. Growth of the stressosome mutants in LBN. A) Growth curve of *V. vulnificus* CMP6 wild-type (green), ΔRSTX (orange) and ΔD1 (purple) in LB + 2.5% NaCl (LBN) at 37°C. The curves are the mean of three biological replicates. For clarity error bars are omitted.

***VvrsbR***

***VvD1***


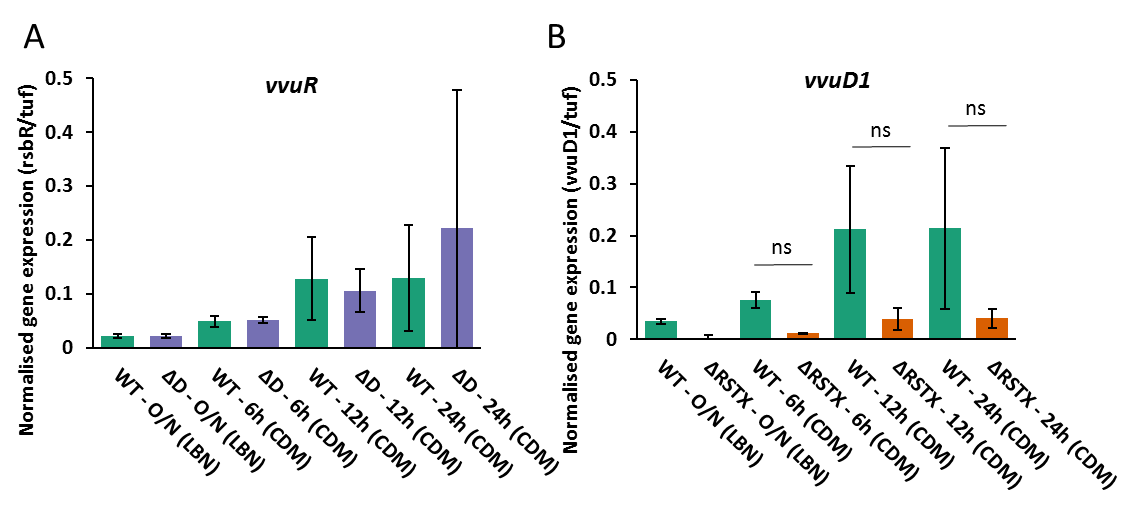


**Figure S4**. Downstream effects of the stressosome mutations on locus transcription. A) *VvrsbR* transcription in *V. vulnificus* CMCP6 wild-type and ΔD1 after overnight growth in LBN and at 6, 12 and 24 h growth in CDM; B) *VvD1* transcription in *V. vulnificus* CMCP6 wild-type and ΔRSTX after overnight growth in LBN and at 6, 12 and 24 h growth in CDM. Normalised gene expression (gene of interest/*tuf*) at each time point is represented. Reported values are the mean +/- standard deviation of three biological replicates. Student's t-test was performed comparing transcription between WT and stressosome mutants at the different time points. All p-values were >0.05.


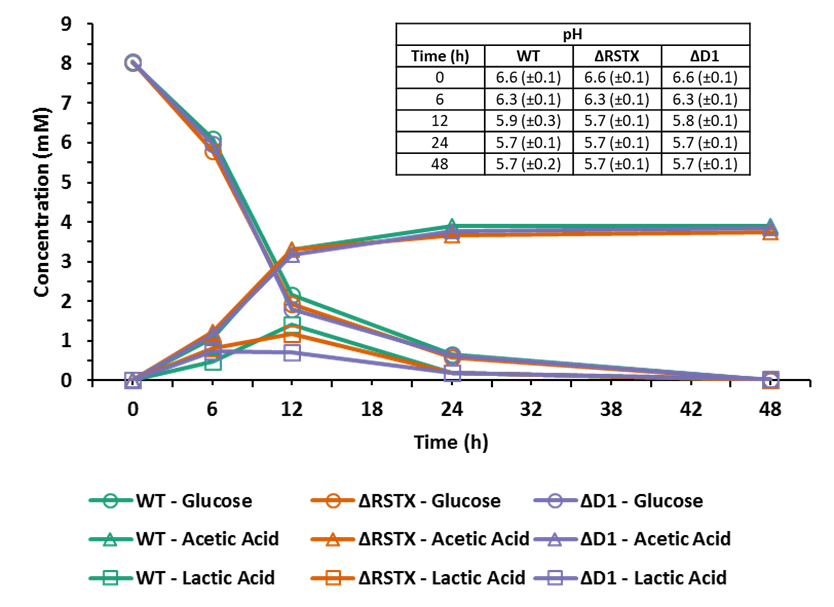


**Figure S5**. Media characterisation during growth in CDM at 37°C of *V. vulnificus* wild-type (green), ΔRSTX (orange) and ΔD1 (purple) mutants. Glucose (circle), acetic acid (triangle) and lactic acid (squares) were quantified through HPLC analysis at 0, 6, 12, 24 and 48 h growth. Inserted table shows pH of the media during growth. The reported values are the mean of three biological replicates.


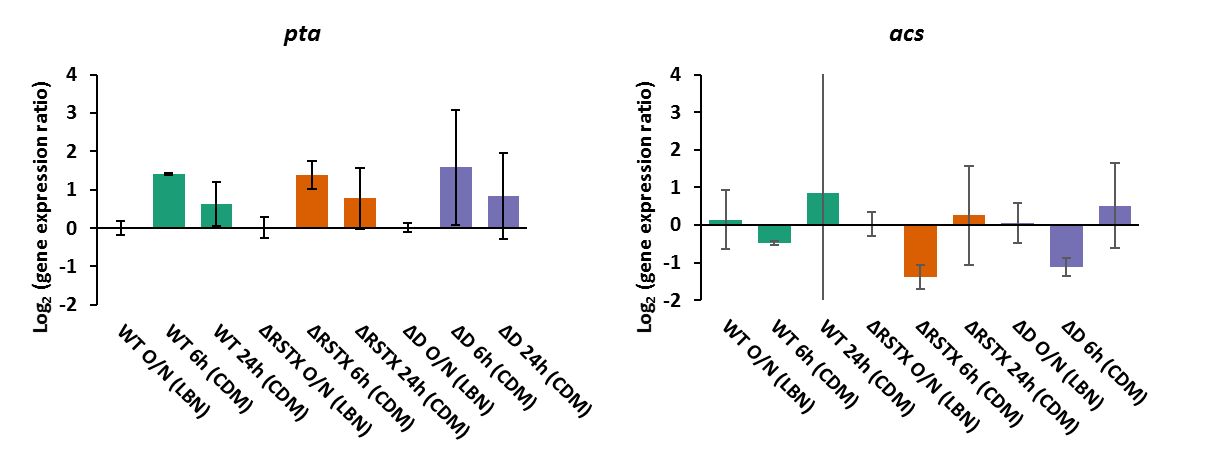


**Figure S6**. Acetate switch *pta* and *acs* gene transcription in *V. vulnificus* CMCP6 wild-type and stressosome mutants after overnight growth (O/N) in LBN and at 6 h and 24 h growth in CDM. The Log_2_ of the ratio between expression at each time point and expression in LBN O/N is represented. Reported values are the mean +/- standard deviation of three biological replicates. Student's t-test was performed comparing expression at different time points, but no significant differences (p-value <0.05) were observed.
